# Supplementary material for: MetaRibo-Seq measures translation in microbiomes
Source: Nat Commun. 2020 Jun 29;11:3268. doi: 10.1038/s41467-020-17081-z (PMC7324362; doi:10.1038/s41467-020-17081-z)
Supplement: Supplementary file 10 — Supplementary Data 7 [file 41467_2020_17081_MOESM10_ESM.zip › File2/Confidence_High_Taxonomy/63489_out.krona.html]

Javascript must be enabled to view this page.

members
magnitude
magnitudeUnassigned
count
unassigned
taxon
rank

63489\_out


SRS017521\_contig\_number\_21147
1
3

superkingdom
2
2

1239
phylum
2

2
class
186801

186802
2
order

2
family
541000

2
genus
216851

853

SRS077024\_contig\_number\_14610SRS149784\_contig\_number\_contig-100\_1328.172890
2
species
